# Hematuria as a risk factor for progression of chronic kidney disease and death: findings from the Chronic Renal Insufficiency Cohort (CRIC) Study

**DOI:** 10.1186/s12882-018-0951-0

**Published:** 2018-06-26

**Authors:** Paula F. Orlandi, Naohiko Fujii, Jason Roy, Hsiang-Yu Chen, L. Lee Hamm, James H. Sondheimer, Jiang He, Michael J. Fischer, Hernan Rincon-Choles, Geetha Krishnan, Raymond Townsend, Tariq Shafi, Chi-yuan Hsu, John W. Kusek, John T. Daugirdas, Harold I. Feldman, the CRIC Study Investigators, Lawrence J. Appel, Alan S. Go, James P. Lash, Panduranga S. Rao, Mahboob Rahman

**Affiliations:** 1 Center for Clinical Epidemiology and Biostatistics, University of Pennsylvania, 824 Guardian Drive, Blockley Hall, Philadelphia, Pennsylvania 19104-6021 USA; 2 Department of Biostatistics, Epidemiology, and Informatics, Perelman School of Medicine at the University of Pennsylvania, Philadelphia, Pennsylvania USA; 3 Hyogo Prefectural Nishinomiya Hospital, Hyogo, Japan; 4 School of Medicine, Tulane University School of Medicine, New Orleans, Louisiana USA; 5 School of Medicine, Wayne State University, Detroit, Michigan USA; 6 Medicine Service, Jesse Brown VA Medical Center, Chicago, Illinois USA; 7 Department of Medicine, University of Illinois at Chicago, Chicago, Illinois USA; 8 Cleveland Clinic Foundation, Case Western Reserve University, Cleveland, Ohio USA; 9 Department of Medicine, University of Pennsylvania, Philadelphia, Pennsylvania USA; 10 John Hopkins University, School of Medicine, Baltimore, Maryland USA; 11 School of Medicine, University of California, San Francisco, California USA; 12 National Institutes of Health, Bethesda, Maryland USA; 13 Renal Division, University of Illinois Hospital and Health Sciences Center, Chicago, Illinois USA

**Keywords:** Hematuria, Epidemiology, CKD, Risk factors, CKD progression, ESRD, Mortality

## Abstract

**Background:**

Hematuria is associated with chronic kidney disease (CKD), but has rarely been examined as a risk factor for CKD progression. We explored whether individuals with hematuria had worse outcomes compared to those without hematuria in the CRIC Study.

**Methods:**

Participants were a racially and ethnically diverse group of adults (21 to 74 years), with moderate CKD. Presence of hematuria (positive dipstick) from a single urine sample was the primary predictor. Outcomes included a 50% or greater reduction in eGFR from baseline, ESRD, and death, over a median follow-up of 7.3 years, analyzed using Cox Proportional Hazards models. Net reclassification indices (NRI) and C statistics were calculated to evaluate their predictive performance.

**Results:**

Hematuria was observed in 1145 (29%) of a total of 3272 participants at baseline. Individuals with hematuria were more likely to be Hispanic (22% vs. 9.5%, respectively), have diabetes (56% vs. 48%), lower mean eGFR (40.2 vs. 45.3 ml/min/1.73 m2), and higher levels of urinary albumin > 1.0 g/day (36% vs. 10%). In multivariable-adjusted analysis, individuals with hematuria had a greater risk for all outcomes during the first 2 years of follow-up: Halving of eGFR or ESRD (HR Year 1: 1.68, Year 2: 1.36), ESRD (Year 1: 1.71, Year 2: 1.39) and death (Year 1:1.92, Year 2: 1.77), and these associations were attenuated, thereafter. Based on NRIs and C-statistics, no clear improvement in the ability to improve prediction of study outcomes was observed when hematuria was included in multivariable models.

**Conclusion:**

In a large adult cohort with CKD, hematuria was associated with a significantly higher risk of CKD progression and death in the first 2 years of follow-up but did not improve risk prediction.

**Electronic supplementary material:**

The online version of this article (10.1186/s12882-018-0951-0) contains supplementary material, which is available to authorized users.

## Background

Hematuria is defined as the presence of red blood cells in the urine originating from the kidney or the urinary tract [1]. Underlying conditions producing hematuria, like diabetes, can be associated with progressive decline in kidney function in the setting of CKD. Also, hematuria, per se may play a mechanistic role in renal disease progression [2]. Hematuria arising from injury in the glomerular filtration barrier, results in passage of red blood cells into the urinary space; promoting oxidative stress, inflammation, and structural damage to the kidney [2–8]. Hematuria can also result from infections, urinary stone disease, tumors, or from other lesions that may obstruct the urinary tract, raising intrarenal pressures, and causing impairment of kidney function [9–11].

Despite the pathophysiological mechanisms relating hematuria to CKD progression, and the low cost and availability of urinary dipstick evaluation, few studies [12, 13] of patients with CKD have examined the association between hematuria and adverse outcomes such as significant loss of kidney function, ESRD, or death [2]. Such studies have focused on smaller and racially-restricted populations, with limited follow-up time and less detailed characterization of participants compared to the Chronic Renal Insufficiency Cohort (CRIC) Study, a prospective study of racially diverse men and women with CKD. In this study, we characterized the association between hematuria assessed at study entry and progression of CKD, ESRD, and all-cause death.

## Methods

### Study population

The Chronic Renal Insufficiency Cohort (CRIC) Study is a multicenter, prospective cohort study which initially enrolled 3939 participants with CKD (eGFR of 20 to 70 ml/min/1.73m^2^ at baseline). The age of enrolled patients ranged from 21 to 74 years, and 48% had a history of diabetes. Details of the CRIC Study design and methods were published previously [14, 15]. Those with polycystic kidney disease, multiple myeloma, glomerulonephritis treated with immunosuppression, or a kidney transplant were ineligible. All participants provided written informed consent. The study protocol was approved by institutional review boards at each center (protocol 807,882 at University of Pennsylvania), and the research was conducted in accordance with the principles of the Declaration of Helsinki.

A total of 3272 CRIC study participants (83%) were tested for hematuria at enrollment. One of the CRIC Study’s seven centers did not implement the dipstick test and all 551 participants from this site were ineligible for this study. The 116 participants who did not undergo testing at sites that tested for hematuria were older, mostly male, and had a higher prevalence of diabetes compared to those tested (Additional file 1).

### Primary exposure

Hematuria was defined as a positive dipstick examination performed once at the time of enrollment.

### Outcomes

The primary outcome was time to a composite of a 50% decline in eGFR from baseline or ESRD. Other outcomes were time to ESRD, defined as date of dialysis initiation or kidney transplantation; and time to death from any cause.

### Covariates

Baseline eGFR was calculated using an internally derived CRIC equation estimating urinary I^125^-iothalamate clearance based on age, sex, race, standardized serum creatinine, and cystatin C and modeled using restricted cubic splines (knots at 30, 45, and 60 ml/min/1.73m^2^) [16].

Baseline albuminuria was log-transformed and also modeled using restricted cubic splines (knots at 30 and 300 mg/day). Other potential confounders included age, gender, race/ethnicity, educational attainment, diabetes mellitus status (defined by fasting glucose> = 126 mg/dl or random blood glucose> = 200 mg/dl or self-reported use of insulin or oral diabetes medication), systolic blood pressure (SBP), body mass index (BMI), use of angiotensin-converting enzyme inhibitor or angiotensin receptor blocker (ACE/ARB), self-reported history of cardiovascular disease (CVD) including coronary artery disease, heart failure, stroke, or peripheral vascular disease.

Other baseline risk factors for CKD included: ankle-brachial index (ABI), hemoglobin, serum uric acid, fibroblast growth factor 23 (FGF-23), parathyroid hormone level (PTH), phosphate, N-terminal pro-B-type natriuretic peptide (NT-pro-BNP), cardiac troponin T, high sensitivity C-reactive protein (CRP), fat-free mass (FFM), insulin resistance index (HOMA-IR), glycated hemoglobin (HbA1c), and plasma lipid levels. Non-normally distributed continuous covariates were categorized into quartiles for inclusion in the time-to-event analysis.

### Statistical analysis

Baseline characteristics of participants with and without hematuria were compared using standard methods for categorical and continuous variables. Multiple imputation was performed for all the covariates that had missing values under the assumption that they were missing at random. A total of 20 imputations was performed using the chained equations method (Additional file 2**).**

### Multivariable modeling approach for explanatory analysis

Using Cox proportional hazards models, we evaluated the association of hematuria with each outcome adjusting for different sets of potential confounders. We employed a series of three nested models: Model 1- unadjusted; Model 2- adjusted for age, sex, race, educational attainment, baseline eGFR, albuminuria, diabetes status, and systolic blood pressure for the renal outcomes (halving of eGFR and/or ESRD); including, as well, smoking status, ankle-brachial index, and history of CVD for the death outcome. Model 3- adjusted for the covariates significantly associated with each outcome at a *P* value ≤ 0.1, when individually added to the Model 2. Variables selected for the final version of Model 3 are presented in Table 2. All models were stratified by study site, allowing for variability in the baseline hazards across centers. For each modeling approach, we explored potential effect modification between hematuria and diabetes, as well as between albuminuria and eGFR level at baseline. Assessments of the validity of the proportional hazards assumption were performed through plots of the log-cumulative survival vs. log survival time (log-log plots), plots of scaled Schoenfeld residuals, and associated tests of non-proportionality.

### Multivariable modeling for clinical risk prediction

We used multivariable logistic regression to assess the incremental benefit of adding hematuria to prediction models. In this modeling strategy, participants were censored at 2 years, and those who were lost to follow-up before 2 years without experiencing the outcome of interest were excluded. To avoid overfitting and to enhance internal validation, we used 5-fold cross-validation. Specifically, we randomly divided our total population into mutually exclusive quintiles, used four groups for training and one group for testing, and repeated this analysis four times, changing the composition of the training and testing data. We built the prediction model using a backward selection strategy, requiring a covariate with the outcome to have a *P* value ≤ 0.1 to be retained. We used backward elimination to ensure the selection of variables with the best predictive capability as a group. All covariates described above were included in this variable selection strategy, independent of their unadjusted association with the outcome.

We assessed model calibration graphically and compared model performance with and without hematuria using C-statistics, net reclassification improvement (NRI) implemented with and without pre-specified risk categories (category-free NRI). The NRI incorporates the fact that a valuable new biomarker will increase the predicted risks or risk categories for events and decrease them for non-events [17–24]. Both estimates may vary from − 1 (assignment in the wrong direction) to + 1(correct direction) [20]. The overall NRI corresponds to the sum of events and non-events NRIs and ranges from − 2 to + 2. For the pre-specified NRI, we used the annualized rates of halving of eGFR and ESRD, ESRD, and death reported previously for the CRIC study as a reference for normal risk ranges [25].

## Results

Among the 3272 CRIC study participants tested with a dipstick exam, 1145 (35%) were found to have hematuria. The prevalence of hematuria increased as the level of eGFR decreased; 24.6% for individuals with an eGFR greater than 60 ml/min/1.73m^2^, 33.3% for those with eGFR between 30 and 60 ml/min/1.73m^2^, and 45.8% for those with eGFR lower than 30 ml/min/1.73m^2^. The prevalence of Hispanic ethnicity, history of diabetes, and high urine albumin levels at baseline were greater among persons with hematuria (Table 1).

**Table 1:** Demographic and clinical characteristics of participants according to the presence or absence of hematuria at baseline

	Dipstick Positive 1145 (35)	Dipstick Negative 2127 (65)	Total 3272 (100)
Demography			
Age (years; mean +/− SD)	55 +/− 12	59+/− 10	57+/− 11
Female Sex (n [%])	470 (41)	935 (44)	1405 (43)
*Racial/ethnic group (n [%])*			
Non-Hispanic White	365 (32)	940 (44)	1305 (40)
Non-Hispanic Black/African American	489 (43)	930 (44)	1419 (43)
Hispanic	254 (22)	204 (10)	458 (14)
Other	37 (3)	53 (2)	90 (3)
*ApoL1 recessive genetic model (n [%])*^*a*^			
0 or 1 copy of APOL1 risk variants	350 (81)	665 (79)	1015 (80)
2 copies of APOL1 risk variants	80 (19)	176 (21)	256 (20)
*Educational attainment (n [%])*			
Less than high school	346 (30)	430 (20)	776 (24)
High school graduate	239 (21)	426 (20)	665 (20)
Some college	319 (28)	607 (29)	926 (28)
College graduate or higher	241 (21)	664 (31)	905 (28)
Anthropometry			
BMI (kg/m^2^; mean +/-SD)	32+/−8	32+/− 8	32+/− 8
*BMI (kg/m*^*2*^*; n [%])*			
< 25	207 (18)	282 (13)	489 (15)
25 to < 30	302 (26)	625 (29)	927 (28)
> =30	633 (55)	1215 (57)	1848 (57)
Abdominal Circumference (cm; mean +/-SD)	105+/− 18	106+/− 17	106+/− 18
Fat free mass [kg; median (IQR)]	61 (50 to 72)	59 (49 to 70)	60 (50 to 71)
Ankle-brachial index< 0.9 (n [%])	209 (19)	326 (16)	535 (17)
Systolic Blood P. (mmHg; mean +/-SD)	134+/− 24	127/− 21	129+/− 22
Diabetes	638 (56)	1014 (48)	1652 (50)
Hypertension	1013 (88)	1878 (88)	2891 (88)
*Tobacco use (n [%])*			
Current smoker	189 (17)	273 (13)	462 (14)
More than 100 cigarettes during lifetime	638 (56)	1180 (55)	1818 (56)
*Cancer (n[%])*			
Any cancer in the last 5 years	68 (6)	158 (7)	226 (7)
Any non-skin cancer in the last 5 years	56 (5)	107 (5)	163 (5)
*Cardiovascular Disease (n [%])*			
Congestive Heart Failure	111 (10)	229 (11)	340 (10)
Peripheral Vascular Disease	103 (9)	137 (6)	240 (7)
Coronary Disease	232 (20)	514 (24)	746 (23)
Cerebrovascular Disesase	124 (11)	215 (10)	339 (10)
Any Cardiovascular Disease	385 (34)	753 (35)	1138 (35)
Renal Function			
eGFR (ml/min/1.73 m2; mean +/-SD)	40+/−15	45+/−16	44+/− 16
*eGFR (ml/min/1.73 m2; n[%]) (CRIC Eq.)*			
< 30	329 (29)	390 (18)	719 (22)
30 to < 40	289 (25)	507 (24)	796 (24)
40 to < 50	239 (21)	480 (23)	719 (22)
50 to < 60	167 (15)	380 (18)	547 (17)
> = 60	121 (11)	370 (17)	491 (15)
Urinalysis			
24H Urine Albumin [g; median (IQR)]	0.50 (0.05 to 1.76)	0.04 (0.0 to 0.26)	0.08 (0.01 to 0.61)
*24H Urine Albumin (n[%])*			
< 30 mg/day	211 (19.54)	951 (46.66)	1162 (37.27)
30 to < 300 mg/day	260 (24.07)	605 (29.69)	865 (27.74)
300 to < 1000 mg/day	224 (20.74)	286 (14.03)	510 (16.36)
> = 1000 mg/day	385 (35.65)	196 (9.62)	581 (18.63)
Other laboratory markers			
Hemoglobin (g/dl; mean+/-SD)	12.3+/−1.9	12.6+/−1.7	12.5+/− 1.8
CalciumT (mg/dl; mean +/-SD)	9.2+/−0.5	9.2+/−0.5	9.2+/−0.5
Phosphate (mg/dl; mean +/-SD)	3.9+/− 0.7	3.7+/− 0.6	3.7+/− 0.7
iPTH [pg/ml; median (IQR)]	63 (38 to 107)	52 (34 to 85)	56 (36 to 92)
FGF23 [RU/ml; median (IQR)]	175 (105 to 284)	141 (95 to 224)	150 (99 to 249)
Vitamin D [ng/ml; median (IQR)]	17.6 (10.8 to 27.2)	22.9 (14.5 to 34)	20.8 (12.8 to 31.7)
Glucose [mg/dl; median (IQR)]	100 (87 to 133)	98 (87 to 123)	98 (87 to 127)
HbA1C [%; median (IQR)]	6.4 (5.6 to 7.9)	6.1 (5.6 to 7.2)	6.2 (5.6 to 7.4)
HOMA^b^[mmol/L*μU/mL; median (IQR)]	4.5 (2.7 to 8.0)	4.1 (2.5 to 7.2)	4.2 (2.6 to 7.5)
Total Cholesterol (mg/dl; mean+/-SD)	191+/−53	179+/−42	183/−46
HDL (mg/dl; mean+/-SD)	46+/−15	47+/−15	47+/−15
LDL (mg/dl; mean+/-SD)	107+/−39	100+/−34	102+/−36
Triglycerides [mg/dl; median (IQR)]	138 (94 to 198)	127 (88 to 183)	130 (91 to 189)
Albumin [g/dl; median (IQR)]	3.8 (3.4 to 4.1)	4.0 (3.7 to 4.3)	3.9 (3.6 to 4.2)
Uric Acid (mg/dl; mean+/-SD)	7.5+/−1.9	7.5+/−1.9	7.5+/−1.9
High sensitivity CRP [mg/l; median (IQR)]	2.8 (1.1 to 6.7)	2.6 (1.1 to 6.6)	2.6 (1.1 to 6.6)
High sens.Troponin T [pg/mL; median (IQR)]	15.7 (7.3 to 34.3)	11.9 (6.0 to 21.6)	12.9 (6.3 to 24.6)
NTproBNP [pg/mL; median (IQR)]	204 (81 to 588)	142 (58 to 370)	162 (66 to 439)
Medication (n [%])			
ACE/ARB	786 (69.3)	1492 (70.7)	2278 (70.2)
Any anti-platelet agent	458 (40.4)	1038 (49.2)	1496 (46.1)
cAMP and Ca modifiers	83 (7.3)	173 (8.2)	256 (7.9)
Cox-1-inhibitor	425 (37.5)	968 (45.9)	1393 (42.9)
Eicosapentaenoic acid	0 (0)	2 (0.09)	2 (0.06)
Heparin	3 (0.26)	0 (0)	3 (0.09)
Vitamin K antagonist	84 (7)	113 (5)	197 (6)

^a^Apol1 recessive genetic model described among a sample of 1271 non-Hispanic black participants. ^b^HOMA: Insulin Resistance Index

Over a median follow-up of 7.3 years 1071 participants experienced halving of eGFR or ESRD, 840 reached ESRD, and 480 died. The crude event rates for participants with hematuria were significantly higher than for those without hematuria (Additional file 3**)**.

**Fig. 1:**
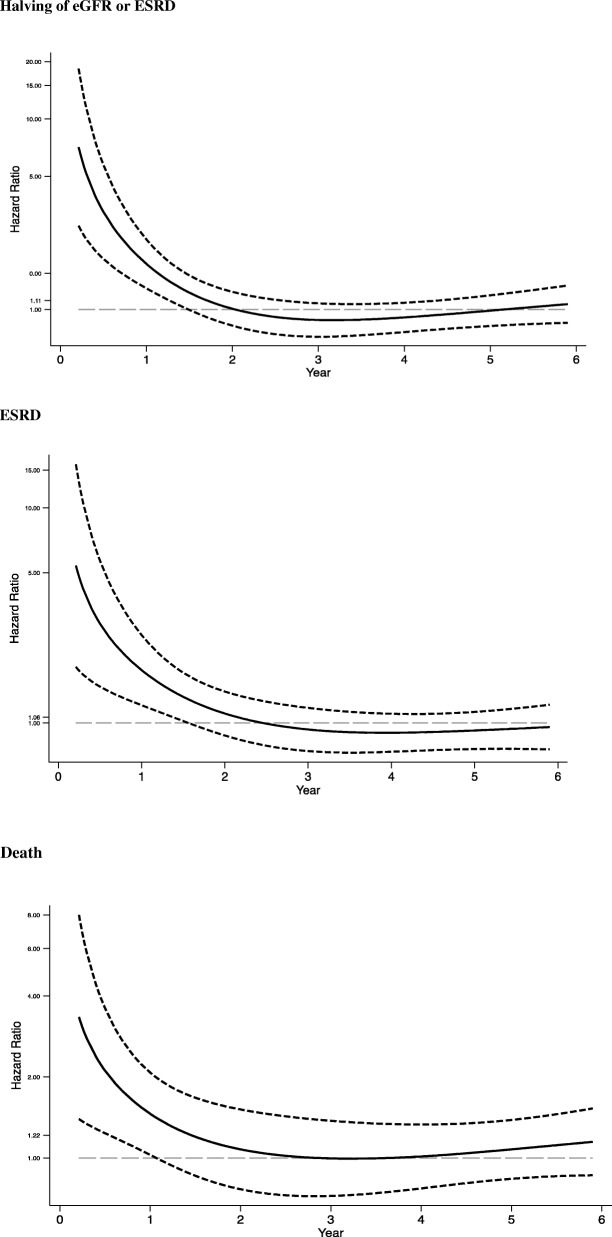
Time-varying Hazard Ratios for CKD progression and death for participants with hematuria compared to those without hematuria. Solid lines indicate the time-varying Hazard Ratios for participants with hematuria compared to those without hematuria for each of the three studied outcomes (Halving of eGFR or ESRD, ESRD, and death). Dashed lines indicate 95% confidence intervals. The natural logarithm of analysis time using restricted cubic splines transformation with 3 knots and its interaction with hematuria was applied to all models above. Model for Halving of eGFR/ESRD was adjusted for age, race, sex, education, eGFR, albuminuria, diabetes, systolic blood pressure, BMI, BNP, HbA1c, FGF23, albumin. Model for ESRD was adjusted for age, race, sex, education, eGFR, albuminuria, diabetes, systolic blood pressure, BMI, BNP, FGF23, serum albumin, high-sensitivity CRP. Model for **Death** was adjusted for age, race, sex, education, eGFR, albuminuria, diabetes, systolic blood pressure, ankle-brachial index, smoking, history of cardiovascular disease, BNP, troponinT, calcium, FGF23, high-sensitivity CRP

The validity of the proportional hazards assumption was not met for any unadjusted model (Additional file 4). Hence, we included an interaction between hematuria and time in all models to further clarify how the associations between hematuria and the outcomes varied over time. We identified significant interactions for all outcomes **(**Table 2**).** This time-trend was consistently observed after multivariable adjustment (Models 2 and 3), revealing significantly higher hazard ratios for renal outcomes and death among participants with hematuria during the first 2 years of follow-up. In Model 3, the hazard ratio for the composite outcome of halving of eGFR or ESRD was 1.74 (95% CI: 1.1 to 2.7, *p* = 0.01) during the first year and 1.4 (95%CI: 1.0 to 1.8, *p* = 0.03) during the second year of follow-up**.** A similar pattern was observed when considering ESRD as the outcome. The hazard ratio for ESRD was 2.2 (95%CI: 1.2 to 4.0, *p* < 0.01) and 1.6 (95% CI:1.1 to 2.3, p = 0.01) in the first year and second year, respectively. The hazard ratios for death also followed the same pattern: 1.9 (95% CI: 1.1 to 3.2, *p* = 0.02) in the first year and 1.75 (95%CI: 1.0 to 3.0, *p* = 0.04) in the second year. The fluctuation of hazard ratios over time was further assessed graphically in models incorporating the continuous form of time (log-transformed, restricted cubic splines with 3 knots) and its interaction with hematuria (Fig. 1). Again, in these models, we observed significantly higher hazard ratios for all outcomes among individuals with hematuria compared to those without hematuria until the end of the second year of follow-up. No interaction with diabetes status, albuminuria, or eGFR at baseline was identified for any of the three outcomes (Additional file 5).

**Table 2:** Hazard ratios of halving of eGFR or ESRD, ESRD, and death for participants with vs. without hematuria according to year of follow-up

	MODEL 1		MODEL 2		MODEL 3	
	HR (95% CI)	p-value	HR (95% CI)	p-value	HR (95% CI)	p-value
Halving of eGFR/ESRD						
year 1	**4.77 (3.15 to 7.25)**	**< 0.001**	**1.89 (1.24 to 2.87)**	**0.003**	**1.74 (1.14 to 2.65)**	**0.01**
year 2	**3.13 (2.37 to 4.13)**	**< 0.001**	**1.45 (1.09 to 1.92)**	**0.01**	**1.38 (1.04 to 1.83)**	**0.03**
year 3	**1.74 (1.29 to 2.34)**	**< 0.001**	0.86 (0.63 to 1.16)	0.32	0.82 (0.60 to 1.11)	0.2
year 4	**2.01 (1.46 to 2.77)**	**< 0.001**	1.01 (0.73 to 1.41)	0.94	0.99 (0.71 to 1.38)	0.94
year 5	**2.35 (1.65 to 3.36)**	**< 0.001**	1.25 (0.87 to 1.79)	0.24	1.22 (0.85 to 1.75)	0.29
after year 5	**1.74 (1.35 to 2.25)**	**< 0.001**	1.07 (0.82 to 1.39)	0.63	1.05 (0.81 to 1.37)	0.7
ESRD						
year 1	**6.79 (3.75 to 12.28)**	**< 0.001**	**2.40 (1.32 to 4.35)**	**0.004**	**2.22 (1.22 to 4.04)**	**0.009**
year 2	**3.85 (2.70 to 5.50)**	**< 0.001**	**1.67 (1.16 to 2.39)**	**0.005**	**1.57 (1.09 to 2.26)**	**0.014**
year 3	**1.61 (1.12 to 2.31)**	**0.01**	0.76 (0.52 to 1.09)	0.13	0.73 (0.51 to 1.06)	0.1
year 4	**1.62 (1.15 to 2.29)**	**0.006**	0.77 (0.54 to 1.10)	0.15	0.75 (0.53 to 1.07)	0.12
year 5	**2.50 (1.73 to 3.63)**	**< 0.001**	1.19 (0.81 to 1.74)	0.37	1.19 (0.81 to 1.74)	0.38
after year 5	**1.76 (1.35 to 2.29)**	**< 0.001**	1.04 (0.79 to 1.36)	0.77	1.06 (0.81 to 1.40)	0.67
Death						
year 1	**2.33 (1.36 to 3.97)**	**0.002**	**2.02 (1.18 to 3.46)**	**0.011**	**1.88 (1.10 to 3.23)**	**0.021**
year 2	**2.07 (1.23 to 3.47)**	**0.006**	**1.88 (1.11 to 3.16)**	**0.018**	**1.75 (1.04 to 2.95)**	**0.035**
year 3	1.14 (0.65 to 2.02)	0.65	1.07 (0.61 to 1.89)	0.81	1.02 (0.58 to 1.80)	0.95
year 4	0.70 (0.38 to 1.28)	0.25	0.66 (0.36 to 1.21)	0.18	0.61 (0.33 to 1.11)	0.11
year 5	1.35 (0.83 to 2.20)	0.23	1.33 (0.81 to 2.17)	0.26	1.19 (0.73 to 1.94)	0.49
after year 5	1.04 (0.74 to 1.46)	0.84	1.12 (0.79 to 1.58)	0.52	1.01 (0.71 to 1.42)	0.97

Halving of eGFR/ESRD: MODEL 1:unadjusted (p-value for the interaction with time:< 0.001); MODEL 2: adjusted for age, race, sex, education, diabetes, eGFR, albuminuria, systolic blood pressure (*p*-value for the interaction with time: 0.025); MODEL 3: adjusted for variables from MODEL 2 and BMI, NT-pro-BNP, HbA1c, FGF23, serum albumin (p-value for the interaction with time: 0.0426);

**ESRD:** MODEL 1: unadjusted (p-value for the interaction with time: < 0.001); MODEL 2: adjusted for age, race, sex, education, diabetes, eGFR, albuminuria, systolic blood pressure (p-value for the interaction with time: 0.001); MODEL 3: adjusted for variables from MODEL 2 and BMI, NT-pro-BNP, FGF23, serum albumin, high-sensitivity CRP (p-value for the interaction with time: 0.0022);

**DEATH:** MODEL 1: unadjusted (p-value for the interaction with time: 0.019); MODEL 2: adjusted for age, race, sex, education, diabetes, eGFR, albuminuria, systolic blood pressure, abi, smoking, cvd (p-value for the interaction with time: 0.0614); MODEL 3: adjusted for variables from MODEL 2 and NT-pro-BNP, High sensitive troponin T, Calcium, FGF23, high-sensitivity CRP (p-value for the interaction with time: 0.0517)

We observed negligible changes in the C-statistics and three-category NRI analysis after incorporating hematuria into statistical models; the free-NRI analysis was the only method to suggest that hematuria may be helpful in the prediction of the studied outcomes (Table 3). Calibration plots for each of the prediction models are depicted in Additional file 6**.** Reclassification tables are presented in Additional file 7**.**

**Table 3:** Summary of prediction improvement assessments comparing models with and without hematuria for halving of eGFR, ESRD and death

	C-statistics			Three categories NRI (95%CI)			Category-Free NRI (95%CI)		
	Hematuria		Increase	Events	Non-events	Overall	Events	Non-events	Overall
	No	Yes							
Halving of eGFR/ESRD^a^	0.897	0.898	0.001	−0.01 (−0.04 to 0.02)	0.01 (−0.01 to 0.02)	0.00 (−0.04 to 0.03)	0.29 (0.18 to 0.41)	0.35 (0.23 to 0.47)	0.64 (0.44 to 0.84)
ESRD^b^	0.929	0.931	0.002	0.01 (−0.04 to 0.05)	0.01 (−0.01 to 0.02)	0.01 (−0.03 to 0.06)	0.41 (0.28 to 0.55)	0.22 (0.01 to 0.42)	0.63 (0.35 to 0.91)
Death^c^	0.770	0.782	0.012	0 (−0.10 to 0.10)	0.01 (−0.02 to 0.04)	0.01 (− 0.10 to 0.12)	0.04 (− 0.14 to 0.22)	0.38 (0.33 to 0.43)	0.42 (0.22 to 0.62)

**Halving of eGFR or ESRD:** model includes: age, race, sex, eGFR, albuminuria, diabetes, systolic blood pressure, BMI, waist circumference, NT-pro-BNP, serum albumin. **ESRD:** model includes age, sex, eGFR, albuminuria, diabetes, waist circumference, NT-pro-BNP, FGF-23, Calcium, iPTH, serum albumin, uric acid, triglycerides. **Death:** model includes BMI, history of CVD, High-sensitive Troponin T, NT-pro-BNP, high sensitivity CRP, use of ACE/ARBs. Categories of predicted probability of the outcome used in the three category NRI: 0 to 10%; > 10 to 15%; > = 15% for halving of eGFR/ESRD; 0 to 5%; > 5 to 10%; > = 10% for ESRD; 0 to 3%; > 3 to 8%; > = 8% for death

## Discussion

Approximately 30% of the studied CKD population had dipstick hematuria at baseline, and the prevalence was greater for lower levels of eGFR and higher levels of albuminuria. During the 24 months following its assessment, hematuria was significantly associated with both CKD progression and death. Despite these associations, incorporating hematuria into risk assessment did not substantively improve prediction of the outcomes of CKD progression or death.

In the general population, the frequency of hematuria is highly variable (0.23 to 17%) and the association between hematuria and renal outcomes in different settings has led to different conclusions [1, 26–29]. In Okinawa, Japan, a study screening about 100,000 individuals found hematuria to be twice as common among those who developed ESRD, compared to those who did not (18 vs. 9%) [29]. In a cohort of more than 170,000 volunteers for health screening in North Carolina, 6.1% of the individuals who developed ESRD had hematuria in contrast to 4.3% of those who did not [30]. Both studies assessed hematuria through dipstick examination, had extended follow-up periods (median of 17 and 25 years, respectively), and detected no association between hematuria and ESRD after multivariable adjustment. In contrast, a study of young adults and adolescents for whom urologic diseases were excluded demonstrated a prevalence of hematuria confirmed by a microscopic exam of only 0.3%. This study, which enrolled 1.2 million individuals, reported a strong association between hematuria and ESRD (HR 18.5; 95%CI:12.4–27.6) after adjustment for age, sex, BMI, and blood pressure [31].

In the CKD population, the prevalence of hematuria has been reported to be approximately 30%, regardless of the method of assessment [12, 13, 32], indicating that, as in our study, hematuria is highly prevalent. However, the association between hematuria and either renal outcomes or death in this population has been infrequently studied. Among those few studies [12, 13, 32] none has demonstrated an overall association between hematuria and CKD progression or death after multivariable adjustment. Recently, 998 participants with CKD, assigned to the placebo treatment of an international trial [12] were studied in a secondary analysis to identify risk factors associated with CKD progression. Thirty-four percent of these participants had hematuria on dipstick examination. Over 4 to 5 years CKD progression occurred in 59 to 76% of the dipstick positive group (depending on the dipstick intensity: trace to 3+), compared to 48% who were dipstick negative. These findings are consistent with ours, but no adjustment for potential confounding was performed, precluding further comparisons. Another prospective cohort study that included 1799 participants with less than 500 mg/g urinary protein [13] demonstrated an association between hematuria (at least 5 red blood cells/high power field) and ESRD (HR 4.41; 95%CI 1.17 to 16.70. An association between hematuria and death was only observed in the subset of the study population who presented at least 5 RBC/HPF and CKD stage 4 (HR: 3.20, 95%CI 1.71 to 5.99). Beyond methodological differences in the assessment of hematuria between this study and ours, the study populations also substantially differed. In particular, compared to the CRIC Study cohort, this study was restricted to non-diabetic older Asians, with more severe CKD (mean eGFR 25.4+/− 15.6 ml/min/1.73m^2^, MDRD equation), and higher levels of proteinuria (881 mg/g, IQR 333–176 mg/g) at baseline.

The association of hematuria with CKD outcomes and death in our study was limited to 2 years of follow-up after multivariable adjustments. One of the possible explanations for this time-limited effect is that the assessment of hematuria was restricted to baseline, and that hematuria did not consistently persist throughout follow-up as many potential causes of hematuria are transient, including IgA nephropathy, acute interstitial nephritis, urinary infections, kidney stones, etc. [2, 3]. This time-varying association between hematuria and progression of kidney disease or death has not been reported previously [12, 13, 33]. Among CKD cohorts that examined the association between hematuria and progression of kidney disease or death, the follow-up time has been shorter than the median 7.3 years in this study. However, all these studies followed participants for approximately 5 years, a duration of follow-up that should have been long enough to detect the interactions with time, but that may not have been explored. Regardless, as we were unable to track the presence of hematuria throughout follow-up we were limited in our capacity to understand this association after the immediate window of time following the assessment for hematuria. Further, previous studies were not able to implement multivariable adjustment for confounding as extensive as implemented in this study. We believe that the consistent association of hematuria observed across all outcomes within the same time interval, after extensive multivariable adjustment, reinforces the strength of our findings.

We hypothesized that individuals with diabetes and hematuria would have a stronger association with CKD progression and death. Even though the prevalence of diabetes was higher among individuals with hematuria, the association with the outcomes did not differ across diabetes status. As no biopsy was performed at baseline, we were unable to directly connect the history of diabetes to the presence of diabetic nephropathy. Many studies suggest that among individuals with CKD and diabetes the presence of micro-hematuria is a signal of non-diabetic nephropathy [34–36]. However, this explanation is quite speculative, as most studies reporting results of biopsies [37, 38] have relied on small restricted groups of patients with atypical clinical presentation [39]. Furthermore, even among individuals with biopsy-proven diabetes the associations between hematuria and faster rates of eGFR decline, ESRD, and death have been inconsistent [38–40].

Despite the strengths of our study, several limitations are noteworthy. First, the evaluation of hematuria was limited to only one dipstick evaluation. Parallel dipstick examinations could have increased our capacity to detect hematuria at baseline, expanding our hematuria positive group. However, had we been able to implement microscopic examination of the urinary sediment to confirm dipstick findings, we may have been able to identify false positive dipstick findings and reduced the hematuria positive group. The American Urological Association, focusing on the screening for malignant lesions in the general population, suggests, based on expert opinion, that the diagnosis of hematuria should be restricted to those individuals who had a positive dipstick confirmed by three or more RBC/HPF on at least two of three properly collected urinary samples [1]. Beyond the boundaries of oncologic screening, a proposed Cochrane meta-analysis comparing screening of general and hospitalized populations with dipstick to urinary microscopic exams found no available randomized trials [41]. The dipstick test has high sensitivity for hematuria (around 85%) [42], but variable specificity (65 to 99%) for renal parenchymal bleeding [1, 43, 44]. Many factors can contribute to false-positive dipstick tests, including menstrual blood, rigorous physical exercise, hemoglobinuria, myoglobinuria, concentrated urine, low specific gravity, and drugs [44]. In light of potential misclassification of hematuria within our study population, we may have diluted the association between hematuria and our clinical outcomes, biasing our findings towards the null. Accordingly, the detection of significant associations likely signal stronger relationships than we observed.

No clinical investigation was prompted by the observation of hematuria in our participants so we were unable to identify its source and further understand if the origin of the hematuria (renal vs. urinary tract) affected the magnitude of the association of dipstick hematuria with outcomes. We could not classify different levels of hematuria or the coexistence of pyuria based on the available data. Also, as for the exposure, covariates were assessed only at baseline and residual confounding could have played a role in our findings given the limitations of our observational study design. Lastly, information was missing on multiple covariates that may have created bias despite our use of multiple imputation.

Few novel risk factors have been proven powerful enough to improve upon the capacity to predict renal outcomes using established markers like proteinuria and eGFR level. The C-statistics obtained for our baseline models are very robust making it difficult to improve upon prediction with the addition of information on hematuria. Our category free-NRI analysis suggested that hematuria may be useful for prediction of renal outcomes and death among CKD patients within the 2 years following its first assessment. However, these findings were not supported by the categorical NRI or by improvement in the C-statistics suggesting that the evaluation of hematuria does not substantially improve prediction of risk of progression of CKD [17, 19–21].

Given the universal access to the dipstick test for hematuria, we understand our finding imply the value of enhanced testing among individuals with CKD. However, analysis of the predictive value of hematuria and our inability to explore the causes of hematuria do not support the conclusion that screening for hematuria in the setting of CKD should be expanded.

## Conclusions

In summary, we observed that hematuria was associated with a significantly greater risk of CKD progression and death within the first 2 years after hematuria ascertainment. Our findings should stimulate further investigation of the impact of hematuria with more specific and sensitive testing strategies. Understanding the pattern of hematuria over time and its relationship to clinical outcomes will be important for fully understanding its predictive value in the setting of CKD.

## Additional files

Additional file 1: Proportional Hazards Plots. Scaled Schoenfeld Residuals Plots by year of follow-up examining if the Proportional Hazards Assumption is valid. (DOCX 26 kb)

Additional file 2: Calibration plots for prediction models. Calibration plots for graphic assessment of prediction models’ validity. (DOCX 21 kb)

Additional file 3: Baseline characteristics of participants and individuals excluded from the study. Baseline characteristics of the 551 CRIC Study participants not included in this analysis. (DOCX 21 kb)

Additional file 4: Multiple Imputation. Number of observations, proportion of missing values, number of observations imputed, and the method used for multiple imputation. (DOCX 13388 kb)

Additional file 5: Incidence Rates. Incidence Rates of Halving of eGFR or ESRD, ESRD, and death overall and according to hematuria status at baseline: (DOCX 21 kb)

Additional file 6: Wald tests for interactions. *p*-values for interactions between albuminuria, diabetes, eGFR and hematuria for the Cox-Proportional Hazards Models detailed in Table 2. (DOCX 637 kb)

Additional file 7: Reclassification table for predicted nonevents and events. Reclassification Table for Nonevents and Events according to Prediction Models with and without Hematuria. (DOCX 25 kb)

## Abbreviations

ABI: Ankle-brachial index
ACE/ARB: Angiotensin-converting enzyme inhibitor or angiotensin receptor blocker
BMI: Body mass index
CKD: Chronic Kidney Disease
CRIC: Chronic Renal Insufficiency Cohort
CRP: high sensitivity C-reactive protein
CVD: Cardiovascular disease, including coronary artery disease, heart failure, stroke, or peripheral vascular disease
eGFR: Estimated glomerular filtration rate
ESRD: End-stage renal disease
FFM: Fat-free mass
FGF-23: Fibroblast growth factor 23
HbA1c: Glycated hemoglobin
HOMA-IR: Insulin resistance index
NRI: Net reclassification index
NT-pro-BNP: N-terminal pro-B-type natriuretic peptide
PTH: Parathyroid hormone level
SBP: Systolic blood pressure
