# Supplementary material for: Hematuria as a risk factor for progression of chronic kidney disease and death: findings from the Chronic Renal Insufficiency Cohort (CRIC) Study
Source: BMC Nephrol. 2018 Jun 26;19:150. doi: 10.1186/s12882-018-0951-0 (PMC6020240; doi:10.1186/s12882-018-0951-0)
Supplement: Supplementary file 1 — Proportional Hazards Plots. Scaled Schoenfeld Residuals Plots by year of follow-up examining if the Proportional Hazards Assumption is valid. (DOCX 26 kb) [file 12882_2018_951_MOESM1_ESM.docx]

**Hematuria as a Risk Factor for Progression of Chronic Kidney Disease and Death:**

Findings from the Chronic Renal Insufficiency Cohort (CRIC) Study

Paula F. Orlandi, MD; Naohiko Fujii, PhD; Jason Roy, PhD; Hsiang-Yu Chen, MS; L. Lee Hamm, MD; James H. Sondheimer, MD; Jiang He, MD, PhD; Michael J. Fischer, MD, MSPH; Hernan Rincon-Choles, MD; Geetha Krishnan, RN, BSN; Raymond Townsend, MD; Tariq Shafi, MBBS, MHS; Chi-yuan Hsu, MD, MSc; John W. Kusek, PhD; John Daugirdas, MD; Harold I. Feldman, MD, MSCE, and the CRIC Study Investigators*

**Additional File 1:** Demographic and clinical characteristics of participants and individuals excluded from the study at baseline.

|  | **Included (3272)** | **Excluded (116)** |
| --- | --- | --- |
| **Demography** | | |
| Age (yr; mean +/- SD) | 57+/-11 | 60+/-11 |
| Female Sex (n [%]) | 1405 (43) | 39 (34) |
| *Racial/ethnic group (n [%])* | | |
| Non-Hispanic White | 1305 (40) | 36 (31) |
| Non-Hispanic Black/ African American | 1419 (43) | 62 (53) |
| Hispanic | 458 (14) | 13 (11) |
| Other | 90 (3) | 5 (4) |
| *ApoL1 recessive genetic model*(n [%])* | |  |
| 0 or 1 copy of APOL1 risk variants | 1015 (80) | 53 (93) |
| 2 copies of APOL1 risk variants | 256 (20) | 4 (7) |
| *Educational attainment (n [%])* |  |  |
| Less than high school | 776 (24) | 21 (18) |
| High school graduate | 665 (20) | 33 (28) |
| Some college | 926 (28) | 35 (30) |
| College graduate or higher | 905 (28) | 27 (23) |
| **Anthropometry** | | |
| BMI (kg/m^2^; mean +/-SD) | 32+/-8 | 32+/-8 |
| *BMI (kg/m^2^; n[%])* |  |  |
| <25 | 489 (15) | 21 (18) |
| 25 to <30 | 927 (28) | 27 (24) |
| >=30 | 1848 (57) | 66 (58) |
| Abdominal Circunf. (cm; mean +/-SD) | 106+/-18 | 107+/-17 |
| Fat free mass [kg; median (IQR)] | 60 (50 to 71) | 60 (52 to 73) |
| Ankle-brachial index<0.9 (n[%]) | 535 (17) | 23 (20) |
| Systolic Blood P. (mmHg; mean +/-SD) | 129+/-22 | 132+/-22 |
| Diabetes | 1652 (50) | 73 (63) |
| Hypertension | 2891 (88) | 97 (84) |
| *Tobacco use (n [%])* |  |  |
| Current smoker | 462 (14) | 22 (19) |
| More than 100 cigarettes during lifetime | 1818 (56) | 71 (61) |
| *Cancer (n[%])* |  |  |
| Any cancer in the last 5 years | 226 (7) | 12 (10) |
| Any non-skin cancer in the last 5 years | 163 (5) | 8 (7) |
| *Cardiovascular Disease (n [%])* |  |  |
| Congestive Heart Failure | 340 (10) | 16 (14) |
| Peripheral Vascular Disease | 240 (7) | 11 (9) |
| Coronary Disease | 746 (23) | 30 (26) |
| Cerebrovascular Disesase | 339 (10) | 16 (14) |
| Any Cardiovascular Disease | 1138 (35) | 49 (42) |
| **Renal Function** | | |
| eGFR (ml/min/1.73m2; mean +/-SD) | 43.6+/-16.2 | 43.9+/-17.9 |
| *eGFR (ml/min/1.73m2; n[%])* |  |  |
| <30 | 719 (22) | 25 (22) |
| 30 to <40 | 796 (24) | 29 (25) |
| 40 to <50 | 719 (22) | 33 (28) |
| 50 to <60 | 547 (17) | 12 (10) |
| >=60 | 491 (15) | 17 (15) |
| **Urinalysis** | | |
| 24H Urine Albumin [g; median (IQR)] | 0.08 (0.01 to 0.6) | 0.09 (0.01 to 0.7) |
| *24H Urine Albumin (g; n[%])* |  |  |
| <30 mg/day | 1162 (37) | 39 (37) |
| 30 to <300 mg/day | 865 (28) | 34 (32) |
| 300 to <1000 mg/day | 510 (16) | 10 (10) |
| >=1000 mg/day | 581 (19) | 22 (21) |
| **Other laboratory measurements** | | |
| Hemoglobin (g/dl; mean+/-SD) | 12.52+/-1.77 | 12.36+/-1.51 |
| CalciumT (mg/dl; mean +/-SD) | 9.25+/-0.46 | 9.24+/-0.39 |
| Phosphate (mg/dl; mean +/-SD) | 3.74+/-0.69 | 3.73+/-0.67 |
| iPTH [pg/ml; median (IQR)] | 56 (35.6 to 92) | 52 (35 to 96) |
| FGF23 [RU/ml; median (IQR)] | 150 (99 to 249) | 175.6 (106 to 301) |
| Vitamin D [ng/ml; median (IQR)] | 20.8 (12.8 to 31.7) | 20.4 (10.9 to 29.5) |
| Glucose [mg/dl; median (IQR)] | 98 (87 to 127) | 112 (90 to 154) |
| HbA1C [%; median (IQR)] | 6.2 (5.6 to 7.4) | 6.8 (5.8 to 8) |
| HOMA [mmol/L*μU/mL; median (IQR)] | 4.2 (2.6 to 7.5) | 4.5 (2.5 to 9.5) |
| Total Cholesterol (mg/dl; mean+/-SD) | 183+/-46 | 178+/-47 |
| HDL (mg/dl; mean+/-SD) | 47+/-15 | 46+/-15 |
| LDL (mg/dl; mean+/-SD) | 102+/-36 | 97+/-38 |
| Triglycerides [mg/dl; median (IQR)] | 130 (91 to 189) | 126 (87 to 188) |
| Albumin [g/dl; median (IQR)] | 3.9 (3.6 to 4.2) | 3.9 (3.6 to 4.2) |
| Uric Acid (mg/dl; mean+/-SD) | 7.5+/-1.9 | 7.6+/-2.0 |
| High sensitivity CRP [mg/l; median (IQR)] | 2.6 (1.1 to 6.6) | 4.0 (1.4 to 8.3) |
| High sens.Troponin T [pg/mL; median (IQR)] | 12.9 (6.3 to 24.6) | 18.4 (9.2 to 30.3) |
| NTproBNP [pg/mL; median (IQR)] | 162 (66 to 439) | 186 (71 to 656) |
|  |  |  |
| **Medication (n [%])** | | |
| ACE/ARB | 2278 (70) | 87 (75) |
| Any anti-platelet agent | 1496 (46) | 58 (50) |
| cAMP and Ca modifiers | 256 (8) | 10 (9) |
| Cox-1-inhibitor | 1393 (43) | 53 (46) |
| Eicosapentaenoic acid | 2 (0.06) | 0 |
| Heparin | 3 (0.1) | 0 |
| Vitamin K antagonist | 197 (6) | 6 (5) |

Participants came from 6 of the 7 CRIC study centers. In one of the 7 centers, screening for hematuria was not performed, and the 551 participants from this center were not included in this analysis. ^a^Apo1 recessive genetic model described among a sample of 1328 non-Hispanic black participants.
