# Supplementary material for: Hematuria as a risk factor for progression of chronic kidney disease and death: findings from the Chronic Renal Insufficiency Cohort (CRIC) Study
Source: BMC Nephrol. 2018 Jun 26;19:150. doi: 10.1186/s12882-018-0951-0 (PMC6020240; doi:10.1186/s12882-018-0951-0)
Supplement: Supplementary file 2 — Calibration plots for prediction models. Calibration plots for graphic assessment of prediction models’ validity. (DOCX 21 kb) [file 12882_2018_951_MOESM2_ESM.docx]

**Hematuria as a Risk Factor for Progression of Chronic Kidney Disease and Death:**

Findings from the Chronic Renal Insufficiency Cohort (CRIC) Study

Paula F. Orlandi, MD; Naohiko Fujii, PhD; Jason Roy, PhD; Hsiang-Yu Chen, MS; L. Lee Hamm, MD; James H. Sondheimer, MD; Jiang He, MD, PhD; Michael J. Fischer, MD, MSPH; Hernan Rincon-Choles, MD; Geetha Krishnan, RN, BSN; Raymond Townsend, MD; Tariq Shafi, MBBS, MHS; Chi-yuan Hsu, MD, MSc; John W. Kusek, PhD; John Daugirdas, MD; Harold I. Feldman, MD, MSCE, and the CRIC Study Investigators*

**Additional File 2:** Multiple Imputation

| **Variable** | **observed (%)** | **imputed (%)** | **method** |
| --- | --- | --- | --- |
| 24 hour urine albumin, g/24h | 3118 (95.3) | 154 (4.7) | predictive mean matching |
| Ankle brachial index | 3215 (98.3) | 57 (1.7) | predictive mean matching |
| FGF-23, RU/ml | 3225 (98.6) | 47 (1.4) | predictive mean matching |
| Total PTH, pg/ml | 3199 (97.8) | 73 (2.2) | predictive mean matching |
| Hba1c, % | 3207 (98) | 65 (2) | predictive mean matching |
| HOMA-IR, mmol/L *uU/ml | 3042 (93) | 230 (7) | predictive mean matching |
| High Sensitive Troponin-T, pg/ml | 3212 (98.2) | 60 (1.8) | predictive mean matching |
| N-terminal pro b-type natriuretic peptide, pg/ml | 3201 (98) | 71 (2) | predictive mean matching |
| High Sensitive C-reactive protein, mg/l | 3261 (99.7) | 11 (0.3) | predictive mean matching |
| Triglycerides, mg/dl | 3261 (99.7) | 11 (0.3) | predictive mean matching |
| Systolic blood pressure, mmHg | 3271 (100) | 1 (0) | linear regression |
| Body mass index, kg/m2 | 3264 (99.8) | 8 (0.2) | linear regression |
| Hemoglobin, g/dl | 3254 (99.4) | 18 (0.6) | linear regression |
| Waist circumference, cm | 3226 (98.6) | 46 (1.4) | linear regression |
| Fat free mass, kg | 3154 (96.4) | 118 (3.6) | linear regression |
| Phosphate, mg/dl | 3209 (98.1) | 63 (1.9) | linear regression |
| Calcium, mg/dl | 3215 (98.3) | 57 (1.7) | linear regression |
| Serum Albumin, g/dl | 3216 (98.3) | 56 (1.7) | linear regression |
| Uric Acid, mg/dl | 3221 (98.4) | 51 (1.6) | linear regression |
| High-density lipoprotein, mg/dl | 3261 (99.7) | 11 (0.3) | linear regression |
| Low-density lipoprotein, mg/dl | 3257 (99.5) | 15 (0.5) | linear regression |
| APOL1 risk alleles | 1271 (90) | 148 (10) | logistic regression |
| Antiplatelet | 3245 (99.2) | 27 (0.8) | logistic regression |
| ACE/ARB | 3245 (99.2) | 27 (0.8) | logistic regression |

A total of 20 multiple imputations were performed applying the chained equations method. Number of observations, proportion of missing values, number of observations imputed, and the method used for imputation are described above. All variables were imputed to complete 3272 observations for each of the 20 imputed datasets, except APOL1 risk alleles, which were imputed within African-American participants only (total 1419).
