# Supplementary material for: Hematuria as a risk factor for progression of chronic kidney disease and death: findings from the Chronic Renal Insufficiency Cohort (CRIC) Study
Source: BMC Nephrol. 2018 Jun 26;19:150. doi: 10.1186/s12882-018-0951-0 (PMC6020240; doi:10.1186/s12882-018-0951-0)
Supplement: Supplementary file 3 — Baseline characteristics of participants and individuals excluded from the study. Baseline characteristics of the 551 CRIC Study participants not included in this analysis. (DOCX 21 kb) [file 12882_2018_951_MOESM3_ESM.docx]

**Hematuria as a Risk Factor for Progression of Chronic Kidney Disease and Death:**

Findings from the Chronic Renal Insufficiency Cohort (CRIC) Study

Paula F. Orlandi, MD; Naohiko Fujii, PhD; Jason Roy, PhD; Hsiang-Yu Chen, MS; L. Lee Hamm, MD; James H. Sondheimer, MD; Jiang He, MD, PhD; Michael J. Fischer, MD, MSPH; Hernan Rincon-Choles, MD; Geetha Krishnan, RN, BSN; Raymond Townsend, MD; Tariq Shafi, MBBS, MHS; Chi-yuan Hsu, MD, MSc; John W. Kusek, PhD; John Daugirdas, MD; Harold I. Feldman, MD, MSCE, and the CRIC Study Investigators*

**Additional File 3:** Incidence Rates of Halving of eGFR or ESRD, ESRD, and death overall and according to hematuria status at baseline:

|  | **(events/1000 person-years)** | **95% CI** | **p-value** |
| --- | --- | --- | --- |
| **Halving of eGFR or ESRD** | |  |  |
| Hematuria negative | 46.7 | 42.9 to 50.8 | <0.001 ^a^ |
| Hematuria positive | 119.6 | 109.9 to 130.2 |  |
| overall | 67.3 | 63.4 to 71.4 | <0.001 ^b^ |
| 15,919 person-years of follow-up, 1071 cases of halving of eGFR or ESRD observed | | | |
| **ESRD** | | | |
| Hematuria negative | 29.1 | 26.4 to 32.1 | <0.001 ^a^ |
| Hematuria positive | 74.6 | 67.9 to 81.9 |  |
| overall | 42.7 | 40.0 to 45.7 | <0.001 ^b^ |
| 19,651 person-years of follow-up, 840 cases of ESRD observed | | | |
| **DEATH** | | | |
| Hematuria negative | 22.8 | 20.4 to 25.4 | 0.023 ^a^ |
| Hematuria positive | 28.3 | 24.3 to 32.9 |  |
| overall | 24.4 | 22.3 to 26.7 | <0.001 ^b^ |
| 19,651 person-years of follow-up, 480 cases of death observed | | | |

^a^ F-test for the association between hematuria and outcomes: p-value<0.05 means that for at least one of the groups (positive or negative for hematuria) the estimated rate of events differs from zero. ^b^ Z-test for the overall rate of events: p-value <0.05 means that the estimated overall rate is significantly different from zero.
