# Supplementary material for: Hematuria as a risk factor for progression of chronic kidney disease and death: findings from the Chronic Renal Insufficiency Cohort (CRIC) Study
Source: BMC Nephrol. 2018 Jun 26;19:150. doi: 10.1186/s12882-018-0951-0 (PMC6020240; doi:10.1186/s12882-018-0951-0)
Supplement: Supplementary file 4 — Multiple Imputation. Number of observations, proportion of missing values, number of observations imputed, and the method used for multiple imputation. (DOCX 13388 kb) [file 12882_2018_951_MOESM4_ESM.docx]

**Hematuria as a Risk Factor for Progression of Chronic Kidney Disease and Death:**

Findings from the Chronic Renal Insufficiency Cohort (CRIC) Study

Paula F. Orlandi, MD; Naohiko Fujii, PhD; Jason Roy, PhD; Hsiang-Yu Chen, MS; L. Lee Hamm, MD; James H. Sondheimer, MD; Jiang He, MD, PhD; Michael J. Fischer, MD, MSPH; Hernan Rincon-Choles, MD; Geetha Krishnan, RN, BSN; Raymond Townsend, MD; Tariq Shafi, MBBS, MHS; Chi-yuan Hsu, MD, MSc; John W. Kusek, PhD; John Daugirdas, MD; Harold I. Feldman, MD, MSCE, and the CRIC Study Investigators*

**Additional File 4:** Plots assessing validity of the Proportional Hazards Assumption for Unadjusted Models

**Halving of eGFR/ESRD**

**
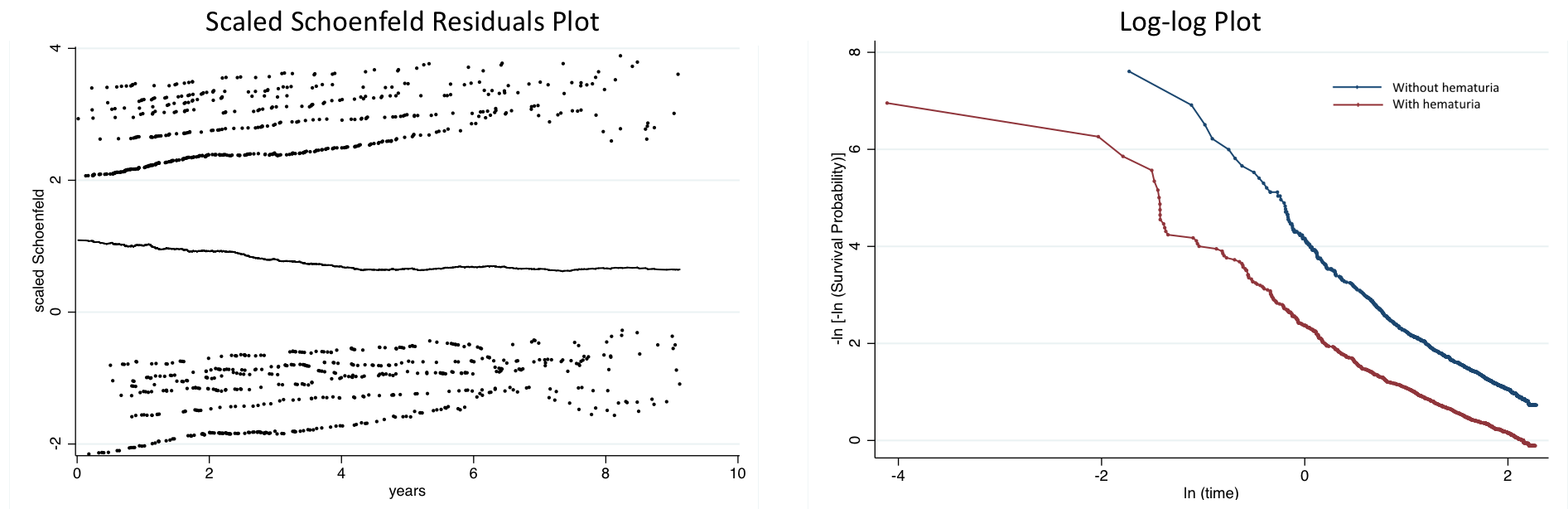
**

**ESRD**


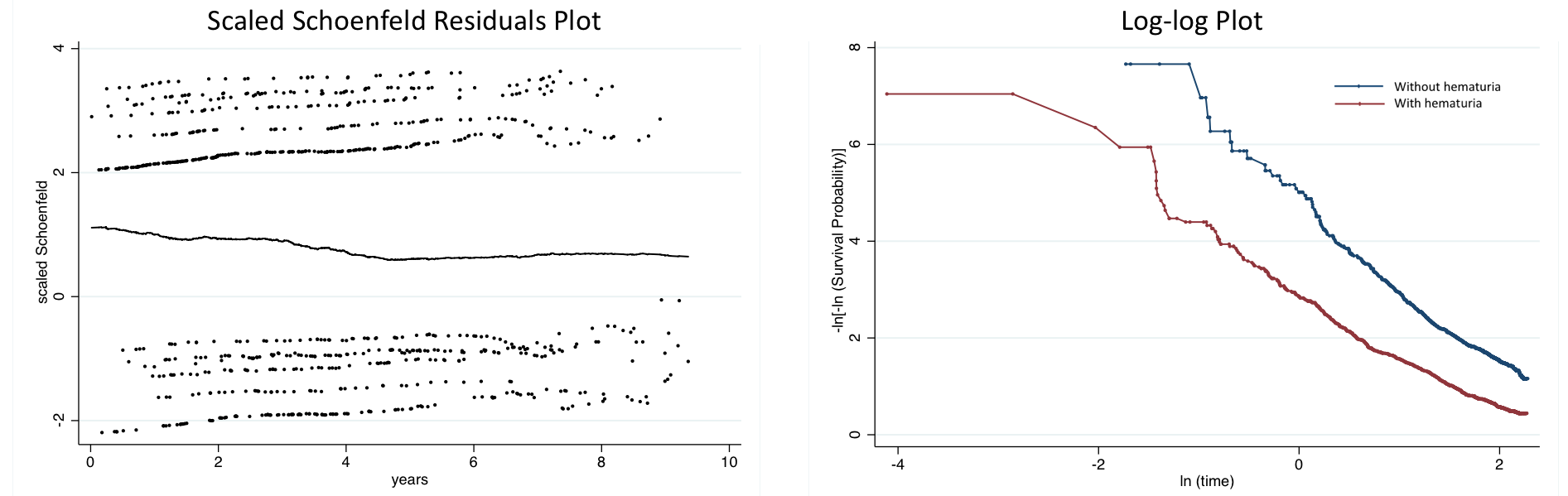


**Death**


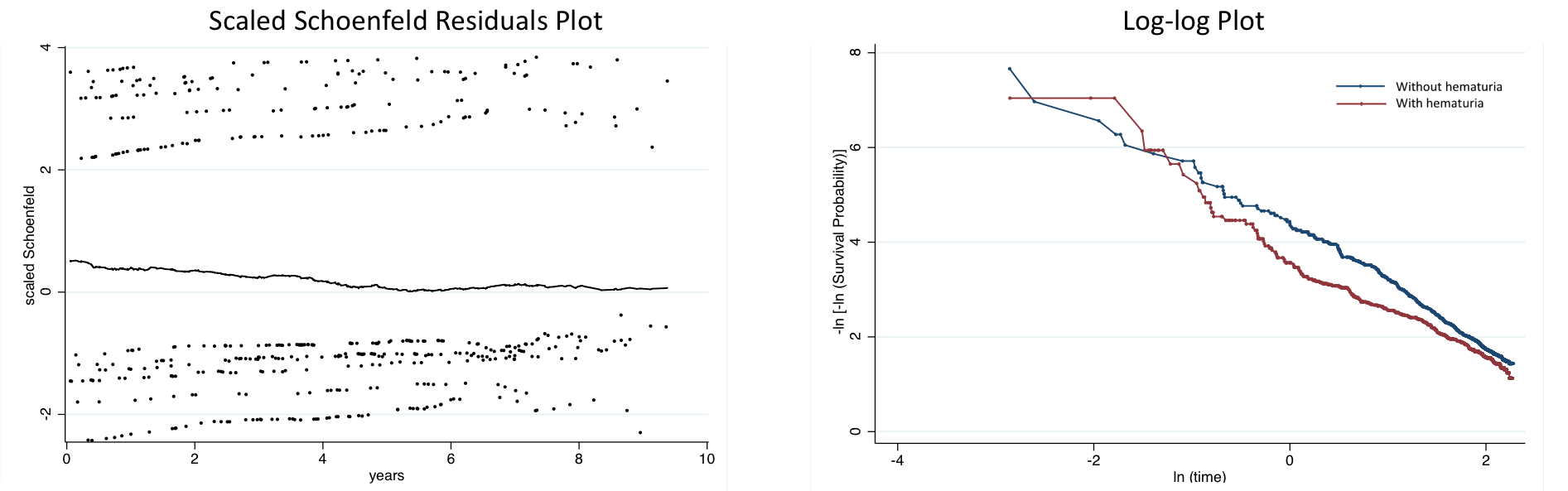


Scaled Schoenfeld Residuals Plots by year of follow-up should present a horizontal line when the Proportional Hazards Assumption is valid. A slight decline can be observed after the second year for all outcomes. Log-log plots [(-log (-log of the survival probability over log (time] should present 2 parallel lines instead of converging (Halving of eGFR/ESRD and ESRD) or crossing and converging (death).
