# Supplementary material for: Hematuria as a risk factor for progression of chronic kidney disease and death: findings from the Chronic Renal Insufficiency Cohort (CRIC) Study
Source: BMC Nephrol. 2018 Jun 26;19:150. doi: 10.1186/s12882-018-0951-0 (PMC6020240; doi:10.1186/s12882-018-0951-0)
Supplement: Supplementary file 5 — Incidence Rates. Incidence Rates of Halving of eGFR or ESRD, ESRD, and death overall and according to hematuria status at baseline: (DOCX 21 kb) [file 12882_2018_951_MOESM5_ESM.docx]

**Hematuria as a Risk Factor for Progression of Chronic Kidney Disease and Death:**

Findings from the Chronic Renal Insufficiency Cohort (CRIC) Study

Paula F. Orlandi, MD; Naohiko Fujii, PhD; Jason Roy, PhD; Hsiang-Yu Chen, MS; L. Lee Hamm, MD; James H. Sondheimer, MD; Jiang He, MD, PhD; Michael J. Fischer, MD, MSPH; Hernan Rincon-Choles, MD; Geetha Krishnan, RN, BSN; Raymond Townsend, MD; Tariq Shafi, MBBS, MHS; Chi-yuan Hsu, MD, MSc; John W. Kusek, PhD; John Daugirdas, MD; Harold I. Feldman, MD, MSCE, and the CRIC Study Investigators*

**Additional File 5:** p-values for interactions between albuminuria, diabetes, eGFR and hematuria for the Cox-Proportional Hazards Models detailed in Table 2.

|  | **MODEL 1** |  | **MODEL 2** |  | **MODEL 3** |
| --- | --- | --- | --- | --- | --- |
| **Halving of eGFR/ ESRD** |  |  |  |  |  |
| interaction between hematuria and albuminuria | 0.76 |  | 0.34 |  | 0.73 |
| interaction between hematuria and diabetes | 0.04 |  | 0.43 |  | 0.35 |
| interaction between hematuria and eGFR | 0.15 |  | 0.31 |  | 0.41 |
|  |  |  |  |  |  |
| **ESRD** |  |  |  |  |  |
| interaction between hematuria and albuminuria | 0.66 |  | 0.19 |  | 0.73 |
| interaction between hematuria and diabetes | 0.04 |  | 0.42 |  | 0.5 |
| interaction between hematuria and eGFR | 0.13 |  | 0.26 |  | 0.37 |
|  |  |  |  |  |  |
| **Death** |  |  |  |  |  |
| interaction between hematuria and albuminuria | 0.17 |  | 0.37 |  | 0.54 |
| interaction between hematuria and diabetes | 0.2 |  | 0.27 |  | 0.45 |
| interaction between hematuria and eGFR | 0.58 |  | 0.43 |  | 0.14 |

**Halving of eGFR/ESRD:** MODEL 1:unadjusted (p-value for the interaction with time:<0.001); MODEL 2: adjusted for age, race, sex, education, diabetes, eGFR, albuminuria, systolic blood pressure (p-value for the interaction with time: 0.025); MODEL 3: adjusted for variables from MODEL 2 and body mass index (BMI), NT-pro-BNP, HbA1c, FGF23, albumin (p-value for the interaction with time: 0.0426); **ESRD:** MODEL 1: unadjusted (p-value for the interaction with time: <0.001); MODEL 2: adjusted for age, race, sex, education, diabetes, eGFR, albuminuria, systolic blood pressure (p-value for the interaction with time: 0.001); MODEL 3: adjusted for variables from MODEL 2 and BMI, NT-pro-BNP, FGF23, serum albumin, high-sensitivity CRP (p-value for the interaction with time: 0.0022);

**DEATH:** MODEL 1: unadjusted (p-value for the interaction with time: 0.019); MODEL 2: adjusted for age, race, sex, education, diabetes, eGFR, albuminuria, systolic blood pressure, Ankle-Brachial index (ABI), smoking, history of cardiovascular disease (CVD (p-value for the interaction with time: 0.0614); MODEL 3: adjusted for variables from MODEL 2 and NT-pro-BNP, high sensitive troponin T, Calcium, FGF23, high-sensitivity CRP (p-value for the interaction with time: 0.0517).
