# Supplementary material for: Hematuria as a risk factor for progression of chronic kidney disease and death: findings from the Chronic Renal Insufficiency Cohort (CRIC) Study
Source: BMC Nephrol. 2018 Jun 26;19:150. doi: 10.1186/s12882-018-0951-0 (PMC6020240; doi:10.1186/s12882-018-0951-0)
Supplement: Supplementary file 6 — Wald tests for interactions. p-values for interactions between albuminuria, diabetes, eGFR and hematuria for the Cox-Proportional Hazards Models detailed in Table 2. (DOCX 637 kb) [file 12882_2018_951_MOESM6_ESM.docx]

**Hematuria as a Risk Factor for Progression of Chronic Kidney Disease and Death:**

Findings from the Chronic Renal Insufficiency Cohort (CRIC) Study

Paula F. Orlandi, MD; Naohiko Fujii, PhD; Jason Roy, PhD; Hsiang-Yu Chen, MS; L. Lee Hamm, MD; James H. Sondheimer, MD; Jiang He, MD, PhD; Michael J. Fischer, MD, MSPH; Hernan Rincon-Choles, MD; Geetha Krishnan, RN, BSN; Raymond Townsend, MD; Tariq Shafi, MBBS, MHS; Chi-yuan Hsu, MD, MSc; John W. Kusek, PhD; John Daugirdas, MD; Harold I. Feldman, MD, MSCE, and the CRIC Study Investigators*

**Additional File 6:** Calibration Plots for Prediction Models

Calibration plots for graphic assessment of prediction models’ validity. The dashed diagonal lines represent the ideal situation where observed and predicted probabilities are equal, while the continuous black lines represent models’ calibration. Each dot depicts the probabilities of the outcome for a hundredth of the whole studied population.
