# Supplementary material for: Hematuria as a risk factor for progression of chronic kidney disease and death: findings from the Chronic Renal Insufficiency Cohort (CRIC) Study
Source: BMC Nephrol. 2018 Jun 26;19:150. doi: 10.1186/s12882-018-0951-0 (PMC6020240; doi:10.1186/s12882-018-0951-0)
Supplement: Supplementary file 7 — Reclassification table for predicted nonevents and events. Reclassification Table for Nonevents and Events according to Prediction Models with and without Hematuria. (DOCX 25 kb) [file 12882_2018_951_MOESM7_ESM.docx]

**Hematuria as a Risk Factor for Progression of Chronic Kidney Disease and Death:**

Findings from the Chronic Renal Insufficiency Cohort (CRIC) Study

Paula F. Orlandi, MD; Naohiko Fujii, PhD; Jason Roy, PhD; Hsiang-Yu Chen, MS; L. Lee Hamm, MD; James H. Sondheimer, MD; Jiang He, MD, PhD; Michael J. Fischer, MD, MSPH; Hernan Rincon-Choles, MD; Geetha Krishnan, RN, BSN; Raymond Townsend, MD; Tariq Shafi, MBBS, MHS; Chi-yuan Hsu, MD, MSc; John W. Kusek, PhD; John Daugirdas, MD; Harold I. Feldman, MD, MSCE, and the CRIC Study Investigators*

**Additional File 7:** Reclassification Table for Nonevents and Events according to Prediction Models with and without Hematuria.

| **A. Halving of eGFR or ESRD** | **Model with Hematuria** | | | | |  |  |
| --- | --- | --- | --- | --- | --- | --- | --- |
|  | 0 -10% |  | 10 - 15% |  | >15% |  | Column Total |
| **Model without hematuria** | N (%) |  | N (%) |  | N (%) |  | N |
|  | **Nonevents (n= 2527)** | | | | |  |  |
| 0 - 10% | 1927 (98) |  | 33 (2) |  | 0 |  | 1960 |
| 10 - 15% | 47 (29) |  | 84 (53) |  | 29 (18) |  | 160 |
| >15% | 0 |  | 33 (8) |  | 374 (92) |  | 407 |
| Row Total | 1974 |  | 150 |  | 403 |  | **2527** |
|  | **Events (n= 345)** | | | | |  |  |
| 0 - 10% | 44 (100) |  | 0 |  | 0 |  | 44 |
| 10 - 15% | 6 (25) |  | 13 (54) |  | 5(21) |  | 24 |
| >15% | 0 |  | 7 (2.5) |  | 270 (97.5) |  | 277 |
| Row Total | 50 |  | 20 |  | 275 |  | **345** |
| **B. ESRD** | **Model with Hematuria** | | | | |  |  |
|  | 0 -5% |  | 5 - 10% |  | >10% |  | Column Total |
| **Model without hematuria** | N (%) |  | N (%) |  | N (%) |  | (%) |
|  | **Nonevents (n= 2878)** | | | | |  |  |
| 0 - 5% | 2301 (99) |  | 33 (1) |  | 0 |  | 2334 |
| 5- 10% | 49 (28) |  | 99 (56) |  | 30 (17) |  | 178 |
| >10% | 0 |  | 46 (13) |  | 320 (87) |  | 366 |
| Row Total (%) | 2350 |  | 178 |  | 350 |  | **2878** |
|  | **Events (n= 226)** | | | | |  |  |
| 0 - 5% | 17 (85) |  | 3 (15) |  | 0 |  | 20 |
| 5 - 10% | 1 (8) |  | 4 (33) |  | 7 (58) |  | 12 |
| >10% | 0 |  | 3 (1.5) |  | 191 (98.5) |  | 194 |
| Row Total (%) | 18 |  | 10 |  | 198 |  | **226** |

| **C. Death** | **Model with Hematuria** | | | | |  |  |
| --- | --- | --- | --- | --- | --- | --- | --- |
|  | 0 -3% |  | 3 - 8% |  | >8% |  | Column Total |
| **Model without hematuria** | N (%) |  | N (%) |  | N (%) |  | (%) |
|  | **Nonevents (n=2878)** | | | | |  |  |
| 0 - 3% | 1723 (93) |  | 133 (7) |  | 0 |  | 1856 |
| 3 - 8% | 161 (24) |  | 445 (67) |  | 61 (9) |  | 667 |
| >8% | 0 |  | 77 (22) |  | 278 (78) |  | 355 |
| Row Total (%) | 1884 |  | 655 |  | 339 |  | **2878** |
|  | **Events (n=112)** | | | | |  |  |
| 0 - 3% | 21 (100) |  | 0 |  | 0 |  | 21 |
| 3 - 8% | 0 |  | 28 (100) |  | 0 |  | 28 |
| >8% | 0 |  | 0 |  | 60 (100) |  | 60 |
| Row Total (%) | 21 |  | 28 |  | 60 |  | **109** |

Each cell contains the number of participants in the corresponding risk categories under models without and with hematuria for each outcome (A, B, and C).
